# Expressive Arts Therapy Combined with Progressive Muscle Relaxation following Music for Perioperative Patients with Gynecological Malignancies: A Pilot Study

**DOI:** 10.1155/2022/6211581

**Published:** 2022-03-29

**Authors:** Xing Liu, Jian-Hua Ren, Sha-Sha Jiang, Yan Tan, Se-Ge Ma, Yan Huang

**Affiliations:** ^1^Department of Obstetrics and Gynecology Nursing, West China Second University Hospital, Sichuan University/West China School of Nursing, Sichuan University, Key Laboratory of Birth Defects and Related Diseases of Women and Children (Sichuan University), Ministry of Education, Chengdu 610041, Sichuan, China; ^2^The First People's Hospital of Liangshan Yi Autonomous Prefecture, Xichang 615000, Sichuan, China

## Abstract

**Objective:**

We aimed to assess the impact of an expressive arts therapy combined with progressive muscle relaxation following music on mental health (anxiety and hope) in patients with gynecological malignancies undergoing surgery.

**Methods:**

This was a nonrandomized controlled trial. Eligible patients had a primary or recurrent gynecological malignancy scheduled to be treated with surgery. The intervention consisted of three sessions (preoperation, postoperation, and predischarge) during the perioperative period. Firstly, before starting the first session of intervention, all patients completed three questionnaires including a Hospital Anxiety and Depression Scale (HADS), a Herth Hope Index (HHI), and a State Anxiety Inventory (SAI), and the intervention group patients also had to complete the SAI questionnaire again after completing the intervention. Secondly, after the second session of intervention, all patients completed the SAI questionnaire, with the intervention group completed the SAI questionnaire before the intervention. Thirdly, after the third session of intervention, all patients completed HHI and SAI questionnaires, with the intervention group completed the SAI questionnaire before the intervention. Also, to subjectively rate the benefit of expressive arts therapy, the intervention group additionally completed a separate, supplemental questionnaire.

**Results:**

A total of 116 patients were enrolled and 110 included in the final analysis. No group differences were found for HHI scores between the intervention and control participants (Cohen's *d* = 0.19, *P*=0.31), although there was a substantial improvement in intervention participants' HHI scores compared to the standard care control participants. There was a statistically significant improvement in intervention participants' SAI from preintervention to postintervention of preoperation (Cohen's *d* = −0.23, *P*=0.002) and postoperation (Cohen's *d* = −0.34, *P* ≤ 0.001). However, no differences were observed for the predischarge period (Cohen's *d* = −0.09, *P*=0.118). Besides, a supplemental questionnaire indicated that 52 (98%) patients felt that expressive arts therapy was beneficial.

**Conclusions:**

Expressive art therapy combined with progressive muscle relaxation under music may be of some effect on alleviating perioperative anxiety in patients with gynecologic malignancies. Therefore, further relevant studies with large samples and multicenters are urgently needed to provide a reliable evidence-based basis for perioperative psychological care of patients with gynecologic malignancies and to promote rapid recovery of patients. It is recommended that further art therapy studies to examine the impact of patient-tailored arts therapy interventions on spiritual well-being in patients with gynecological malignancies, especially in the perioperative period.

## 1. Introduction

Gynecological cancers are amongst the most common malignancies affecting women, including cervical, ovarian, uterine, endometrial, vaginal, fallopian tube, placental, and vulval cancer [1]. Recently, the International Agency for Research on Cancer (IARC) today of the World Health Organization released the latest estimates on the global burden of cancer in 2020. It showed that among the top ten cancers with new cases of women in the world, 600,000 (6.5%) were cervical cancer, 420,000 (4.5%) were endometrial cancer, and 310,000 (3.4%) were ovarian cancer; Among the top ten cancers with cancer deaths in the world, 340,000 (7.7%) were cervical cancer and 210,000 (4.7%) were ovarian cancer [2].

Receiving the diagnosis of gynecological malignancy was emotionally devastating [3]. Once diagnosed, the treatment of gynecological malignancies was mainly based on surgery, supplemented by chemotherapy and radiotherapy [4, 5]. The surgery itself was a medical stressor, combined with the specificity of the surgical site (radical surgery often removed the uterus, ovaries, fallopian tubes, or other organs representing female characteristics), patients with gynecological malignancies were more vulnerable to psychological symptoms such as anxiety and depression during the perioperative period [6]. What's worse, although the aim of surgical for gynecological cancer is to cure and to improve survival rates [7], years after, women have to deal with fundamental changes and challenges concerning their physical, mental, and psychosocial well‐being because of surgical morbidity, chemotherapy toxicities, loss of fertility, changes in body image, sexual concerns, and altered relationships [8–10]. Meanwhile, the reproductive system has a special significance for women, it is the bond that holds the relationship between the sexes together, and its absence may lead to marital instability and even family breakdown, which directly threatens family life [11]. Consequently, compared with patients with nongynecological malignancies, patients with gynecological malignancies may have more mental stress. Anxiety and depression, as negative emotions, also affected the occurrence, development, and prognosis of the disease [12].

Art therapy, as an alternative, nonpharmacological treatment, has become increasingly popular in many fields in recent years. Expressive arts therapy was defined as the use of art, music, dance/movement, drama, poetry/creative writing, play, and sand tray within psychotherapy, counseling, rehabilitation, or health care [13]. As it possesses several specific characteristics not always found in strictly verbal therapies, expressive arts therapies added a unique dimension to psychotherapy and counseling, including, but not limited to, self-expression, active participation, imagination, and mind-body connections. Expressive therapies had been incorporated into various programs as an element of both the foundational and supportive treatment [14], achieving significant results in many other fields [15–18]. Music is a powerful and effective medium which can assist in reducing anxiety, pain, and stress. Music therapy was feasible and well accepted in terminally ill cancer patients during specialized inpatient palliative care [19]. Both listening to recorded monochord sounds and practising progressive muscle relaxation were useful and comparable for gynecologic oncological patients during chemotherapy [20].

However, there were few studies on the effectiveness of expressive arts therapy in patients with gynecological malignancies and even fewer in the perioperative period [21, 22]. Our study aimed to assess the impact of expressive arts therapy combined with progressive muscle relaxation under music on anxiety and hope in patients with gynecological malignancies undergoing surgery. We hypothesized that expressive arts therapy combined with progressive muscle relaxation under music would improve the patients' anxiety and hope during the perioperative period as measured by the State Anxiety Inventory (SAI) and the Herth Hope Index (HHI).

## 2. Materials and Methods

### 2.1. Ethics Approval

We designed and conducted the study following the guidelines for ethical principles outlined in the Declaration of Helsinki [23]. This study was approved by the Ethics Committee of West China Second University Hospital, Sichuan University, with the reference Medical Research 2020 Lun approvalno. (160) on August 1, 2020. Also, all participants provided oral informed consent.

### 2.2. Patients Selection

This was a prospective, nonrandomized controlled trial evaluating the effects of expressive arts therapy on anxiety and hope among patients with gynecological malignancies undergoing surgery. We recruited patients diagnosed with gynecological malignancies hospitalized at the Jinjiang District of the West China Second University Hospital, Sichuan University, from May 2020 to January 2021, and scheduled to undergo surgery to participate in this study. All patients with gynecological malignancies undergoing surgery were invited to participate in the study. After a patient expressed interest in participating in the study, the research assistant (Sha-Sha Jiang) approached the patient to discuss the participation. This study was exploratory naturally to understand any benefit from implementing expressive arts therapy combined with progressive muscle relaxation following music. Therefore, the patients' decisions to participate in the activities were voluntary. Informed and agreed to participate in this study, the patients were enrolled into the intervention group or the control group based on their decisions.

#### 2.2.1. Inclusion Criteria

Patients meeting all of the following inclusion criteria were encouraged to participate in the study: (1) diagnosis with any type or stage of gynecological malignancies to undergo surgery; (2) age of 18 years or older; (3) consciousness was clear to communicate normally; and (4) informed and agreed to participate in this study.

#### 2.2.2. Exclusion Criteria

Exclusion criteria included as follows: (1) patients combined with other severe physical or mental diseases; (2) patients with intellectual or cognitive impairment; (3) patients combined with severe postoperative complications; (4) participants withdrew from the study at any point before the end of the study; and (5) the vacant content of filled questionnaire ≥20%.

### 2.3. Intervention

**Figure 1:**
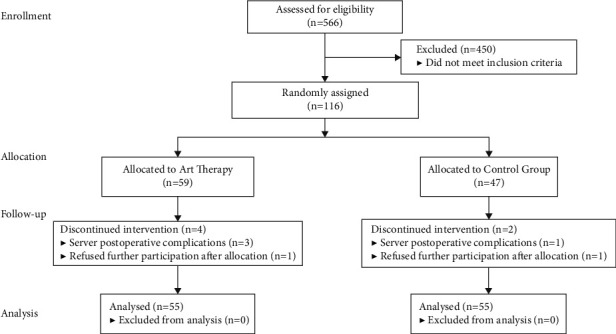
Flow diagram of participants' recruitment.

Based on the participants' decisions, the 116 included patients were divided into the intervention group (59) and the control group (57). At the end of the study, four patients fell off in the intervention group and two in the control group. The flow diagram of participant recruitment is shown in Figure 1. The control group used conventional perioperative care. In addition to conventional perioperative care, the intervention group systematically applied expressive arts therapy with three consecutive interventions: preoperation, postoperation, and predischarge. The expressive arts therapy was performed by nurses who had successfully completed coursework approved for 60 hours of training credits in the expressive arts therapy and obtained expressive arts therapy certificate. All patients were not allowed to receive psychological interventions or psychotropic medication except the intervention during the study to avoid contamination. The expressive arts therapy sessions were further described in detail below.

#### 2.3.1. Conventional Perioperative Care

The conventional perioperative used for the control group was as follows: perioperative routine nursing, psychological nursing, symptom management, and health education, including encouraging and comforting patients, establishing good nurse-patient relationships, and explaining the knowledge of the disease.

#### 2.3.2. Expressive Arts Therapy Intervention

The expressive arts therapy intervention consisted of three consecutive sessions: preoperation(T1), postoperation(T2), and predischarge(T3), duration was left to the patients' decision.

The first session: power and blessing. The intervention was carried out one day before the operation. It was group therapy that patients were encouraged to dance and handcraft under music. The handicraft creations included various forms of beads stringing, rope weaving, clay making, etc. Patients could freely choose one or more materials to create their works to express their surgical blessings. Upon completion, participants shared the content and meaning of their work to express their feelings before surgery.

Before starting the intervention, all patients completed three questionnaires comprising of a Hospital Anxiety and Depression Scale (HADS), a Herth Hope Index (HHI), and a State Anxiety Inventory (SAI). Also, the intervention group were also invited to complete the SAI questionnaire again immediately after the completing intervention.

The second session: enjoying the tranquil moment. The intervention was carried out one day after the patient returned to the ward after the operation one-on-one at the bedside. The patient listened to music under the guidance of the nurse's instruction, with relaxation. Patients were guided to follow the music and perform progressive muscle relaxation practice to empty and release themselves wholeheartedly.

Before the intervention, the intervention group were invited to complete the SAI questionnaire. Also, after the intervention, all patients completed the SAI questionnaire.

The final session: i am discharged today. The intervention was carried out one day before the discharge one-to-one at the bedside. The patients were provided with colored pens and paper to draw and free write to record the current feelings, such as expectations for the future, hospitalization experience, saying to family members and medical staff, and the vision of life after discharge.

Before the intervention, the intervention group were invited to complete the SAI questionnaire. Also, after the intervention, all patients completed HHI and SAI questionnaires and the intervention group additionally completed a separate, supplemental questionnaire to rate the benefit of expressive arts therapy (a scale of 1 to 10 : 1 being not beneficial and 10 being extremely beneficial) [22].

### 2.4. Assessments

#### 2.4.1. Hospital Anxiety and Depression Scale (HADS)

The HADS was developed by Zigmond and Snaith [24] in 1983 to provide clinicians and scientists with a reliable, valid, and practical tool for identifying and quantifying anxiety and depression in medical patients [25]. The scale consisted of 14 items of seven rated anxiety and another seven depression (with possible ranges of 0–21 for each subscale), making it easy to administer and well-accepted [26, 27].

#### 2.4.2. Herth Hope Index (HHI)

The HHI [28] was developed by Herth in 1992, to measure a global, nontime oriented sense of hope based on the multidimensional concept of hope as theorized by Dufault and Martocchio. It consisted of three dimensions: (1) inner sense of temporality and future, (2) inner positive readiness and expectancy, and (3) interconnectedness with self and others. The items were scored on a 4-point Likert-type scale (1 = strongly disagree to and 4 = strongly agree) and calculated by summing each item's score, with items 3 and 6 reverse-scored. Scores range from 4 to 48, and a higher value signifies a higher hope. The HHI had been widely used in research and clinical practice in different cultural settings for various health areas [29, 30].

#### 2.4.3. State Anxiety Inventory (SAI)

The State-Trait Anxiety Inventory (STAI) was developed by Charles Spielberger and his co-authors. It was a brief, reliable, valid, self-report, 40-item psychological test for adults in research and clinical settings to assess feelings of immediate anxiety that an individual feels at the current moment (state anxiety, SAI) and dispositional anxiety (trait anxiety, TAI) [31]. It consisted of a 20-item state anxiety scale and a 20-item trait anxiety scale. Higher scores reflected higher levels of anxiety.

### 2.5. Statistical Analyses

The descriptive statistics were used to create a demographic profile of the intervention and control groups. Baseline equivalence between the two groups was examined by use of chi-square and independent samples *t*-test. Since data met the criteria for parametric statistics, the differences in the scores of HHI between the intervention and control groups were evaluated by use of an independent samples *t*-test, and a paired samples *t*-test was applied to examine the within-group differences for the intervention group for SAI over the three consecutive sessions (preoperation, postoperation, and predischarge). All statistical computations were performed using IBM SPSS Statistics version 23.0 and Microsoft Excel 2016. The *P* value of less than 0.05 was considered a statistically significant difference. The effect size was calculated (expressed as Cohen's d), with ‘small,' ‘medium,' and ‘large' effects (*d* = 0.2, 0.5, and 0.8, respectively) [32].

## 3. Results

### 3.1. Participant Characteristics

A total of 116 patients who consented to participate in the study were enrolled and divided into either the intervention group (*n* = 59) or the control group (*n* = 57) and completed baseline measurements. At the end of the study, four patients fell off in the intervention group and two in the control group. A total of 110 were included in the final analysis. There were no significant differences between the intervention group and the control group, which was an indication that patients in the two groups were comparable at baseline for sociodemographic and clinical characteristics (Table 1).

### 3.2. Quantitative Results

There were no statistically significant differences between the intervention group and control group participants for HHI scores (Cohen's d = 0.19, *P*=0.31), although there was a substantial improvement in the intervention group participants' HHI scores compared to the control group participants. However, the patients' HHI scores in both groups improved from preintervention to postintervention of the three consecutive sessions with statistically significant differences (Table 2).

**Figure 2:**
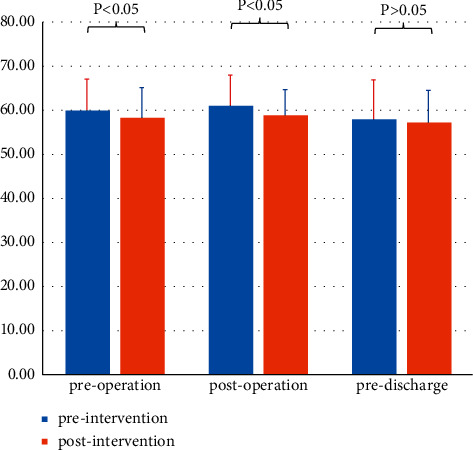
Comparison of SAI scores before and after intervention for the three sessions in the intervention group.

There was a statistically significantly greater improvement in the intervention group participants' SAI from preintervention to postintervention of the first session (preoperation, T1) and the second session (postoperation, T2), with *p* value 0.002 and ≤ 0.001, respectively. However, there were no statistically significant differences from preintervention to postintervention of the final session (predischarge, T3), with a *p* value of 0.118 (Table 3 and Figure 2).

Besides, 55 patients completed the supplemental questionnaire that asked how beneficial the session to her on a scale of 1 to 10. The supplemental questionnaire indicated that 94.5% (52/55) of the patients felt that expressive arts therapy was beneficial (score >5), and the remained (3/55) rated the expressive arts therapy as 5.

## 4. Discussion

Gynecological cancer patients bore a tremendous psychological and physiological burden, and the surgery itself as a source of stressor may place an even heavier psychological burden on the patient. Therefore, medical staff must explore and evaluate other avenues to improve psychological well-being for patients with gynecologic malignancies in the perioperative period. Overall, our study showed that expressive arts therapy combined with progressive muscle relaxation following music administered by nurses were effective in reducing patients' immediate anxiety. That further consolidated the findings that art therapies can help patients with malignancies alleviate anxiety levels and alter the mood [33–36]. Therefore, these expressive arts therapies can be considered effectively adjunct to traditional treatments for gynecological cancer patients with anxiety during the perioperative period.

### 4.1. Expressive Arts Therapy Combined with Progressive Muscle Relaxation following Music for HHI

Hope is a belief in the perseverance of life, regardless of the future outcome. Hope can alleviate pain and physical function obstruction [37]. In the healing process, hope is a powerful strategy for patients to cope with cancer as it helps the patient cope with the shackles of the disease and the threat of death [38]. Also, there is a significant correlation between the level of personal hope, personal health condition, and psychological changes [39]. Therefore, studies about the hope level of cancer patients are of great clinical significance.

This study evaluated the efficacy of expressive arts therapy combined with progressive muscle relaxation following music for hope improvement in gynecological cancer patients during the perioperative period. The HHI scores improved in both groups and slightly more improved in the intervention group, but this difference was not statistically significant. There was no evidence of a treatment effect for expressive arts therapy combined with progressive muscle relaxation following music on overall hope in gynecological cancer patients during the perioperative period. Hope is complicated and strongly associated with hopelessness, rare studies had addressed hope in women recently diagnosed with gynecologic cancer and suggested nurses in a unique position to continue to practice hope-inspiring nursing to assist patients in fighting homelessness [40, 41]. Three explanations were possible for the study findings. Firstly, surgery is the most prominent means to eradicate gynecological cancers. The level of hope of early-stage gynecological cancer patients improved after surgery [42]. It was consistent with the research result that although cervical cancer patients suffered from physical and psychological distress, most were still confident and hopeful about the future [43]. Secondly, the intervention effect was underpowered due to the short duration of perioperative hospital stay. Hope is complicated. Many factors affected a patient's level of hope, including interpersonal interactions, value evaluation, achievable goals, positive personality traits, and spiritual beliefs [44]. Therefore, understanding hope from a disease perspective may allow healthcare staff to develop strategies to better foster hope so that patients can cope with disease positively [45]. Thirdly, the study was underpowered due to the study sample, a larger study sample, significant between-group differences may be obtained.

### 4.2. Expressive Arts Therapy Combined with Progressive Muscle Relaxation following Music for SAI

One significant finding of this study was that expressive arts therapy combined with progressive muscle relaxation following music had a significant effect on preoperative and postoperative current anxiety in gynecological cancer patients, confirming those conclusions regarding the possible ability of art therapy to alleviate anxiety in cancer patients, which was consistent with the results of other studies [46, 47]. Including music therapy as a complementary modality with cancer surgery may help manage pre-operative anxiety in a safe, effective, time-efficient, and enjoyable way [48]. For the immediate predischarge anxiety in patients with gynecologic malignancies, although the expressive arts therapy alleviated the current anxiety, there was no statistically significant difference before and after the intervention.

Estimated that 25% to 80% of patients hospitalized for proposed surgical treatment experienced preoperative anxiety [49, 50]. Patients often feel nervous and uneasy about the disease, anesthesia, and surgery due to their lack of surgery experience. The state of anxiety can affect the communication and mutual trust between the doctor and patient. Besides, it can result in a negative impact on the patient's preoperative preparation and postoperative rehabilitation [51, 52]. Although surgical anxiety may be managed by administering larger dosages of anxiolytic drugs, these drugs can depress circulation and respiration, making nondrug alternatives particularly attractive [53]. Adequate and effective communication between medical staff and patients before surgery can alleviate patients' anxiety to a certain extent, but traditional preoperative conversation methods and health education had a limited role in improving patients' anxiety state [54]. Patients' predischarge anxiety may be closely related to readiness for hospital discharge. Discharge readiness was influenced by many factors, including patient factors [55], hospital factors [56], and social factors [57]. Therefore, for the predischarge anxiety of patients with gynecologic malignancy undergoing surgery, nurses need to comprehensively understand the patients' readiness for discharge to make targeted interventions to effectively alleviate the patients' predischarge anxiety and thus improve the patients' quality of life after hospital discharge. Therefore, expressive arts therapy based on patient needs for perioperative patients' anxiety states is crucial. However, evidence of art therapy in gynecological perioperative cancer patients was quite insufficient [21].

### 4.3. Expressive Arts Therapy Combined with Progressive Muscle Relaxation following Music for Patient Experience

In our study, all but three patients receiving expressive arts therapy combined with progressive muscle relaxation following music evaluated the overall experience as very positive, which further confirmed the current conclusions for the benefit of art therapy [22, 35]. Expressive arts therapy can be therapeutic in an expressing and ventilative manner, and it should be encouraged into the treatment practices for our patients. As reflected in our study, expressive arts therapy combined with progressive muscle relaxation following music resulted in a significant impact that should not be underestimated on perioperative anxiety in patients with gynecological malignancies.

### 4.4. Limitations

The findings reported in the present study must be considered in light of the study design and interventional approach. Some limitations of this study included a small sample size. Since we had a limited number of eligible patients at only an institution, a randomized sample was not feasible. That limited the applicability of the study across all gynecological perioperative cancer patients. Another limitation was that nurses and participants could not be blinded due to the treatment's nature, which may have introduced bias into the results. Besides, for the questionnaire evaluating the benefit of expressive arts therapy intervention, though told that this survey was anonymous and to respond truthfully, patients may have felt that they needed to respond positively to the treatment due to social desirability bias.

### 4.5. Implications for Future Studies

In oncology, art therapy as a supportive care intervention was a relatively new and studies used a variety of study designs, making it hard to draw conclusions [58, 59]. What was worse, studies in this field contained some limitations, including our study. Therefore, more randomized controlled trials with larger sample sizes are needed to establish the evidence of expressive arts therapy's effectiveness for patients with gynecological malignancies, particularly in the perioperative period. The expressive arts therapy could possibly help decrease symptoms of anxiety and depression in adult cancer patients, and further research using stringent methods was urgently needed because of the heterogeneity of the interventions and limited methodological quality of the previous studies [60]. The findings of our study may provide a basis for future research with regard to evaluating the use of expressive arts therapy in the management of anxiety and hope in various surgical populations with gynecological malignancies.

## 5. Conclusions

This study did not find statistically significant differences in hope between participants in the expressive arts therapy combined with progressive muscle relaxation following music group and the standard care control group, although hope in both groups improved from preintervention to postintervention with statistically significant differences. In the expressive arts therapy combined with progressive muscle relaxation following music group, participants' immediate anxiety was statistically significantly improved from preintervention to postintervention in preoperation and postoperation periods. Nevertheless, there were no statistically significant differences in the pre-discharge period. Besides, most participants felt that expressive arts therapy was beneficial. It is recommended that further expressive arts therapy studies examine the impact of patient-tailored arts therapy interventions on spiritual well-being in patients with gynecological malignancies, especially in the perioperative period.

## Figures and Tables

**Table 1:** Descriptive statistics for sociodemographic and clinical characteristics.

Characteristics	Intervention group (*n* = 55)	Control group (*n* = 55)	*P* value
Age(years), mean ± sd	48.18 ± 10.82	45.29 ± 10.97	0.17
Religious faith, n(%)			
No	52 (94.5)	52 (94.5)	0.66
Yes	3 (5.5)	3 (5.5)	
Education, n(%)			
Elementary school and below	13 (23.6)	12 (21.8)	0.94
Junior High School	17 (30.9)	19 (34.5)	
High school	8 (14.5)	10 (18.2)	
Junior college	13 (23.6)	10 (18.2)	
Undergraduate	4 (7.3)	4 (7.3)	
Marital status, n(%)			
Married	48 (87.3)	49 (89.1)	0.37
Single	3 (5.5)	1 (1.8)	
Divorced	4 (7.3)	3 (5.5)	
Widowed	0 (0)	2 (3.6)	
Place of residence, n(%)			
Rural	15 (27.3)	22 (40.0)	0.35
Town	19 (34.5)	17 (30.9)	
City	21 (38.2)	16 (29.1)	
Medical insurance, n(%)			
Yes	53 (96.4)	54 (98.2)	0.50
No	2 (3.6)	1 (1.8)	
Residential pattern, n(%)			
Living alone	1 (1.8)	1 (1.8)	0.60
Living with family members	53 (96.4)	54 (98.2)	
Living with others	1 (1.8)	0 (0)	
Diagnosis, n(%)			
Cervical cancer	30 (54.5)	32 (58.2)	0.46
Ovarian cancer	9 (16.4)	11 (20.0)	
Endometrial cancer	12 (21.8)	5 (9.1)	
Vulvar cancer	1 (1.8)	2 (3.6)	
Fallopian tube cancer	0 (0)	1 (1.8)	
Pelvic malignant tumor	3 (5.5)	4 (7.3)	
Chemotherapy history, n(%)			
Yes	9 (16.4)	10 (18.2)	0.50
No	46 (83.6)	45 (81.8)	
Radiotherapy history, n(%)			
Yes	0 (0)	3 (5.5)	0.12
No	55 (100)	52 (94.5)	
HADS, mean ± sd			
Anxiety scores	5.76 ± 3.37	5.98 ± 3.39	0.67
Depression scores	4.36 ± 3.49	4.24 ± 3.36	0.58

HADS, Hospital Anxiety and Depression Scale; sd, standard deviation.

**Table 2:** Comparison of HHI scores between the two groups (mean ± sd; effect size).

Groups	Preintervention (T1)	Postintervention (T3)	t Value	*P* value	Effect size (Cohen's d)	T3-T1
Expressive arts therapy group (*n* = 55)	22.87 ± 3.45	29.60 ± 2.29	12.64	≤0.001	2.28	6.73 ± 3.95
Standard care control group (*n* = 55)	24.00 ± 3.72	29.98 ± 1.86	12.14	≤0.001	2.02	5.99 ± 3.65
t Value	−1.648	−0.960				1.028
*P* value	0.10	0.34				0.31
Effect size (Cohen's d)	−0.31	−0.18				0.19

HHI, Herth Hope Index; sd, standard deviation; T1, treatment 1 (preoperation); T3, treatment 3 (predischarge).

**Table 3:** Comparison of SAI for the three consecutive sessions within the intervention group (mean ± sd; effect size).

The three sessions	Preintervention	Postintervention	t Value	*P* value	Effect size (Cohen's d)
Preoperation (T1)	59.91 ± 7.16	58.25 ± 6.88	3.33	*P*=0.002	−0.23
Postoperation (T2)	61.02 ± 6.97	58.82 ± 5.86	4.92	*P* < 0.001	−0.34
Predischarge (T3)	57.93 ± 8.98	57.20 ± 7.32	1.59	*P*=0.118	−0.09

S-AI, State Anxiety Inventory; T1, treatment 1 (preoperation); T2, treatment 2 (postoperation); T3, treatment 3 (predischarge).

## Data Availability

The authors maintain full control of all the primary data and agree to allow the journal to review the data upon request.

## References

[1] Sankaranarayanan R., Ferlay J. (2006). Worldwide burden of gynaecological cancer: the size of the problem. *Best Practice & Research Clinical Obstetrics & Gynaecology*.

[2] World Health Organization International Agency for Research on Cancer *Latest Global Cancer Data: Cancer Burden Rises to 19.3 Million New Cases and 10.0 Million Cancer Deaths in 2020*.

[3] O Connor A. P., Wicker C. A., Germino B. B (1990). Understanding the cancer patient’s search for meaning. *Journal of Cancer Nursing*.

[4] Fabian A., Krug D., Alkatout I. (2020). Radiotherapy and its intersections with surgery in the management of localized gynecological malignancies: a comprehensive overview for clinicians. *Journal of Clinical Medicine*.

[5] Karadayi K., Yildiz C., Karakus S. (2016). Cytoreductive surgery and perioperative intraperitoneal chemotherapy for gynecological malignancies: a single center experience[J]. *European Journal of Gynaecological Oncology*.

[6] Reis N., Beji N. K., Coskun A. (2010). Quality of life and sexual functioning in gynecological cancer patients: results from quantitative and qualitative data. *European Journal of Oncology Nursing*.

[7] Shah C. A., Beck T., Liao J. B., Giannakopoulos N. V., Veljovich D., Paley P. (2017). Surgical and oncologic outcomes after robotic radical hysterectomy as compared to open radical hysterectomy in the treatment of early cervical cancer. *Journal of Gynecologic Oncology*.

[8] Tabano M., Condosta D., Coons M. (2002). Symptoms affecting quality of life in women with gynecologic cancer. *Seminars in Oncology Nursing*.

[9] Bahri N., Tohidinik H. R., Fathi Najafi T., Larki M., Amini T., Askari Sartavosi Z. (2016). Depression following hysterectomy and the influencing factors. *Iranian Red Crescent Medical Journal*.

[10] Sekse R. J. T., Dunberger G., Olesen M. L., Østerbye M., Seibæk L. (2019). Lived experiences and quality of life after gynaecological cancer-An integrative review. *Journal of Clinical Nursing*.

[11] Dyson G. J., Thompson K., Palmer S., Thomas D. M., Schofield P. (2012). The relationship between unmet needs and distress amongst young people with cancer. *Supportive Care in Cancer*.

[12] Yeh Y. C, Huang S. F, Lu C. H (2019). Correlation among anxiety, depression, and quality of life in women with gynecologic cancer. *Hu Li Za Zhi*.

[13] Malchiodi C. A. (2005). *Expressive Therapies: History, Theory, and Practice[M]*.

[14] Hoffmann B. (2016). The role of expressive therapies in therapeutic interactions; art therapy - explanation of the concept. *Trakia Journal of Science*.

[15] Visser M., du Plessis J. (2015). An expressive art group intervention for sexually abused adolescent females. *Journal of Child and Adolescent Mental Health*.

[16] Klagsbrun J., Rappaport L., Speiser V. M. (2008). Focusing and expressive arts therapy as a complementary treatment for women with breast cancer. *Journal of Creativity in Mental Health*.

[17] Nan J. K. M., Lau B. H.-P., Szeto M. M. L., Lam K. K. F., Man J. C. N., Chan C. L. W. (2018). Competence enhancement program of expressive arts in end-of-life care for health and social care professionals: a mixed-method evaluation. *American Journal of Hospice and Palliative Medicine*.

[18] Siegel J., Iida H., Rachlin K., Yount G. (2016). Expressive arts therapy with hospitalized children: a pilot study of Co-creating healing sock creatures. *Journal of Pediatric Nursing*.

[19] Kordovan S., Preissler P., Kamphausen A., Bokemeyer C., Oechsle K. (2016). Prospective study on music therapy in terminally ill cancer patients during specialized inpatient palliative care. *Journal of Palliative Medicine*.

[20] Lee E.-J., Bhattacharya J., Sohn C., Verres R. (2012). Monochord sounds and progressive muscle relaxation reduce anxiety and improve relaxation during chemotherapy: a pilot EEG study. *Complementary Therapies in Medicine*.

[21] Fu W., Huang Y., Liu X., Ren J., Zhang M. (2020). The effect of art therapy in women with gynecologic cancer: a systematic review. *Evidence-based Complementary and Alternative Medicine*.

[22] Wiswell S., Bell J. G., McHale J., Elliott J. O., Rath K., Clements A. (2019). The effect of art therapy on the quality of life in patients with a gynecologic cancer receiving chemotherapy. *Gynecologic Oncology*.

[23] World Medical Association (2013). World medical association declaration of Helsinki. *JAMA*.

[24] Zigmond A. S., Snaith R. P. (1983). The hospital anxiety and depression scale. *Acta Psychiatrica Scandinavica*.

[25] Herrmann C. (1997). International experiences with the Hospital Anxiety and Depression Scale-A review of validation data and clinical results. *Journal of Psychosomatic Research*.

[26] Mackenzie L. J., Carey M. L., Sanson-Fisher R. W., D’Este C. A., Paul C. L., Yoong S. L. (2014). Agreement between HADS classifications and single-item screening questions for anxiety and depression: a cross-sectional survey of cancer patients. *Annals of Oncology*.

[27] Hartung T. J., Friedrich M., Johansen C. (2017). The hospital anxiety and depression scale (HADS) and the 9-item patient health questionnaire (PHQ-9) as screening instruments for depression in patients with cancer. *Cancer*.

[28] Herth K. (1992). Abbreviated instrument to measure hope: development and psychometric evaluation. *Journal of Advanced Nursing*.

[29] Chan K. S., Li H. C. W., Chan S. W.-C., Lopez V. (2012). Herth Hope Index: psychometric testing of the Chinese version. *Journal of Advanced Nursing*.

[30] Rustøen T., Lerdal A., Gay C., Kottorp A. (2018). Rasch analysis of the Herth hope Index in cancer patients. *Health and Quality of Life Outcomes*.

[31] Sydeman S. (2018). State-trait anxiety inventory. *Encyclopedia of Personality and Individual Differences*.

[32] Nakagawa S., Cuthill I. C. (2007). Effect size, confidence interval and statistical significance: a practical guide for biologists. *Biological Reviews*.

[33] Puetz T. W., Morley C. A., Herring M. P. (2013). Effects of creative arts therapies on psychological symptoms and quality of life in patients with cancer. *JAMA Internal Medicine*.

[34] Puig A., Lee S. M., Goodwin L., Sherrard P. A. D. (2006). The efficacy of creative arts therapies to enhance emotional expression, spirituality, and psychological well-being of newly diagnosed Stage I and Stage II breast cancer patients: a preliminary study. *The Arts in Psychotherapy*.

[35] Saw J. J., Curry E. A., Ehlers S. L. (2018). A brief bedside visual art intervention decreases anxiety and improves pain and mood in patients with haematologic malignancies. *European Journal of Cancer Care*.

[36] Doll M., Roshon S. G., Stone E. R., Butler R. S. (2017). Evaluation of art therapy on mood, anxiety, and pain levels in patients with cancer undergoing chemotherapy treatment. *Journal of Clinical Oncology*.

[37] Saleh U. S., Brockopp D. Y. (2001). Hope among patients with cancer hospitalized for bone marrow transplantation. *Cancer Nursing*.

[38] Rustøen T (1995). Hope and quality of life, two central issues for cancer patients: a theoretical analysis. *Cancer Nursing*.

[39] Stoner M. H., Keampfer S. H. (1985). Recalled life expectancy information, phase of illness and hope in cancer patients. *Research in Nursing & Health*.

[40] Hammer K., Mogensen O., Hall E. O. C. (2009). Hope as experienced in women newly diagnosed with gynaecological cancer. *European Journal of Oncology Nursing*.

[41] Hammer K., Hall E. O. C., Mogensen O. (2013). Hope pictured in drawings by women newly diagnosed with gynecologic cancer. *Cancer Nursing*.

[42] Ebina Y., Mikami M., Nagase S. (2019). Japan Society of Gynecologic Oncology guidelines 2017 for the treatment of uterine cervical cancer. *International Journal of Clinical Oncology*.

[43] Li L.-R., Lin M.-G., Liang J. (2017). Effects of intrinsic and extrinsic factors on the level of hope and psychological health status of patients with cervical cancer during radiotherapy. *Medical Science Monitor*.

[44] Herth K. (1990). Fostering hope in terminally-ill people. *Journal of Advanced Nursing*.

[45] Beng T. S., Xin C. A., Ying Y. K. (2020). Hope in palliative care: a thematic analysis. *Journal of Palliative Care*.

[46] Czamanski-Cohen J., Weihs K. L. (2016). The bodymind model: a platform for studying the mechanisms of change induced by art therapy. *The Arts in Psychotherapy*.

[47] Holmqvist G. R, Roxberg S, Larsson I (2017). What art therapists consider to be patients inner change and how it mayappear during art therapy. *Arts in Psychotherapy*.

[48] Bradley Palmer J., Lane D., Mayo D., Schluchter M., Leeming R. (2015). Effects of music therapy on anesthesia requirements and anxiety in women undergoing ambulatory breast surgery for cancer diagnosis and treatment: a randomized controlled trial. *Journal of Clinical Oncology*.

[49] Hellstadius Y., Lagergren J., Zylstra J. (2016). Prevalence and predictors of anxiety and depression among esophageal cancer patients prior to surgery. *Diseases of the Esophagus*.

[50] Ruis C., Wajer I. H., Robe P., van Zandvoort M. (2017). Anxiety in the preoperative phase of awake brain tumor surgery. *Clinical Neurology and Neurosurgery*.

[51] Britteon P., Cullum N., Sutton M. (2017). Association between psychological health and wound complications after surgery. *British Journal of Surgery*.

[52] John M. (2009). Managing anxiety in the elective surgical patient. *British Journal of Nursing*.

[53] Walther-Larsen S, Diemar V, Valentin N (1988). Music during regional anesthesia A reduced need of sedatives. *Regional Anesthesia and Pain Medicine*.

[54] Kalogianni A., Almpani P., Vastardis L., Baltopoulos G., Charitos C., Brokalaki H. (2016). Can nurse-led preoperative education reduce anxiety and postoperative complications of patients undergoing cardiac surgery?. *European Journal of Cardiovascular Nursing*.

[55] Mabire C., Coffey A., Weiss M. (2015). Readiness for Hospital Discharge Scale for older people: psychometric testing and short form development with a three country sample. *Journal of Advanced Nursing*.

[56] Chan C. W. H., Chan D. N. S., So W. K. W., Chen J. M. T., Sit J. W. H. (2015). Innovative health promotion program on breast cancer screening for ethnic minority women in Hong Kong. *Journal of Obstetric, Gynecologic, and Neonatal Nursing*.

[57] Wallace A. S., Perkhounkova Y., Bohr N. L., Chung S. J. (2016). Readiness for hospital discharge, health literacy, and social living status. *Clinical Nursing Research*.

[58] Geue K., Goetze H., Buttstaedt M., Kleinert E., Richter D., Singer S. (2010). An overview of art therapy interventions for cancer patients and the results of research. *Complementary Therapies in Medicine*.

[59] Wood M. J. M., Molassiotis A., Payne S. (2011). What research evidence is there for the use of art therapy in the management of symptoms in adults with cancer? A systematic review. *Psycho-Oncology*.

[60] Bosman J. T., Bood Z. M., Scherer-Rath M. (2020). The effects of art therapy on anxiety, depression, and quality of life in adults with cancer: a systematic literature review. *The Effects of Art Therapy on Anxiety, Depression, and Quality of Life in Adults with Cancer: A Systematic Literature review[J] Supportive Care in Cancer*.

